# Novel Primate-Specific Genes, RMEL 1, 2 and 3, with Highly Restricted Expression in Melanoma, Assessed by New Data Mining Tool

**DOI:** 10.1371/journal.pone.0013510

**Published:** 2010-10-20

**Authors:** Josane F. Sousa, Raul Torrieri, Rodrigo R. Silva, Cristiano G. Pereira, Valeria Valente, Erico Torrieri, Kamila C. Peronni, Waleska Martins, Nair Muto, Guilherme Francisco, Carla Abdo Brohem, Carlos G. Carlotti, Silvya S. Maria-Engler, Roger Chammas, Enilza M. Espreafico

**Affiliations:** 1 Departamento de Biologia Celular e Molecular e Bioagentes Patogênicos, Faculdade de Medicina de Ribeirão Preto, Universidade de São Paulo, Ribeirão Preto, São Paulo, Brazil; 2 Departamento de Cirurgia e Anatomia, Faculdade de Medicina de Ribeirão Preto, Universidade de São Paulo, Ribeirão Preto, São Paulo, Brazil; 3 Clinical Staff, Molecular Biology Group, Pathology Division, AC Camargo Hospital Melanoma Group, São Paulo, São Paulo, Brazil; 4 Departamento de Radiologia e Instituto do Câncer do Estado de São Paulo, Faculdade de Medicina, Universidade de São Paulo, São Paulo, São Paulo, Brazil; 5 Departamento de Análises Clínicas e Toxicológicas, Faculdade de Ciências Farmacêuticas, Universidade de São Paulo, São Paulo, São Paulo, Brazil; University of Pennsylvania, United States of America

## Abstract

Melanoma is a highly aggressive and therapy resistant tumor for which the identification of specific markers and therapeutic targets is highly desirable. We describe here the development and use of a bioinformatic pipeline tool, made publicly available under the name of EST2TSE, for the *in silico* detection of candidate genes with tissue-specific expression. Using this tool we mined the human EST (Expressed Sequence Tag) database for sequences derived exclusively from melanoma. We found 29 UniGene clusters of multiple ESTs with the potential to predict novel genes with melanoma-specific expression. Using a diverse panel of human tissues and cell lines, we validated the expression of a subset of three previously uncharacterized genes (clusters Hs.295012, Hs.518391, and Hs.559350) to be highly restricted to melanoma/melanocytes and named them RMEL1, 2 and 3, respectively. Expression analysis in nevi, primary melanomas, and metastatic melanomas revealed RMEL1 as a novel melanocytic lineage-specific gene up-regulated during melanoma development. RMEL2 expression was restricted to melanoma tissues and glioblastoma. RMEL3 showed strong up-regulation in nevi and was lost in metastatic tumors. Interestingly, we found correlations of RMEL2 and RMEL3 expression with improved patient outcome, suggesting tumor and/or metastasis suppressor functions for these genes. The three genes are composed of multiple exons and map to 2q12.2, 1q25.3, and 5q11.2, respectively. They are well conserved throughout primates, but not other genomes, and were predicted as having no coding potential, although primate-conserved and human-specific short ORFs could be found. Hairpin RNA secondary structures were also predicted. Concluding, this work offers new melanoma-specific genes for future validation as prognostic markers or as targets for the development of therapeutic strategies to treat melanoma.

## Introduction

Melanoma is an aggressive tumor marked by high metastatic potential and drug resistance [1], [2]. In the last decades its incidence worldwide has increased considerably [3]. Thus, great interest exists in the identification of genes of melanoma-specific expression that may lead to new markers to monitor the disease status or to new therapeutic targets.

Tissue-restricted expression is a desirable property for candidate genes as therapeutic targets in cancer, since their function could be inhibited without damaging normal tissues, their promoters could be used to enhance expression of cell death-inducing proteins in tumor cells, and tumor-specific proteins could serve as targets for immunotherapy or site-specific delivery of antitumor agents.

Besides the annotation of human genes, expressed sequence tags (ESTs) have also been used for the identification of tissue-specific genes [4], [5], including genes differentially expressed in normal and tumor tissues [6], and those encoding cancer/testis tumor antigens, characterized by their predominant expression in germ cells, trophoblast cells, and tumor tissues [7]. More recently, EST data and its associated UniGene information were used to create a database of genes preferentially expressed in 30 tissues [8], [9].

In this work, we report the identification of a group of UniGene clusters containing multiple ESTs derived exclusively from melanoma. We also describe the scheme of the *in silico* pipeline used for detection of tissue-specific genes based on the EST database and make the software generated publicly available. Moreover, we show experimental validation of an unprecedentedly highly restricted melanoma expression profile for a subset of three genes besides some clues to their functions according to correlations between gene expression and clinical data. Finally, based on sequence analysis, we present the exon/intron organization of these genes and putative gene products.

## Results

### Identification of 29 UniGene clusters represented by expressed sequences exclusively detected in melanoma libraries

With the aim of identifying genes with melanoma restricted expression we developed a simple *in silico* pipeline that then was implemented as a new online tool, EST2TSE (Table S1). The tool allows the mining of all available human EST data in a search for candidate genes with tissue restricted expression. Although our tool is simpler than tools of similar function, such as Gene Library Summarizer (GLS) [10] from the Cancer Genome Anatomy Project-CGAP, UniGene Digital Differential Display – DDD [11] and the Tissue-specific Gene Expression and Regulation-TiGER database [12], it allows a more straightforward search for specific terms, for example, some tumor types or subtypes, since we can use as keyword any of the terms associated to the cell or tissue type in the GenBank reports (Table S1). Actually, the TiGER database does not allow a search for melanoma-specific genes, since it presents a pre-defined list of tissue names that does not include melanoma, instead, only offers the term “skin”. Similar to GLS from CGAP, EST2TSE detects candidate genes with tissue- or condition-restricted expression (unique genes, represented by sequences found only in the selected tissue/condition), in contrast to UniGene Digital Diferential Display – DDD and TiGER that detect genes with enriched expression.

Using this pipeline tool we identified 215 UniGene clusters composed of ESTs detected only in melanoma libraries. Of these, 177 clusters were composed of a single EST per cluster and thus were removed to minimize the chance of selecting artifacts or genes with very low expression. The other 38 clusters with multiple ESTs or complete cDNA sequences underwent additional analysis by BLAST and BLAT alignments, which eliminated nine additional clusters that matched cDNA sequences from tissues other than melanoma. Six clusters containing ESTs from other tumor types (Hs.382776, Hs.407538, Hs.586239, Hs.637822, Hs.650135) or from testis (Hs.570414), besides those from melanoma libraries, were included, as they might represent tumor antigens. As summarized in Table S2, of the 29 clusters selected, 19 have coding potential based on the occurrence of deduced amino acid sequences in GenBank or as predicted by the CPC software [13], and most have ORFs encoding less than 200 aa. Among the predicted proteins there are 5 putative nucleic acid-binding proteins, a tubulin annotated as tubulin beta4Q, and 13 proteins with no characterized domains. Among the predicted nucleic acid-binding proteins there is a member (SSX-5) of a known family of cancer-testis antigens. Also, one ORF (Hs.570414) encodes a transmembrane protein as predicted by Phobius [14]. The clusters Hs.551051 and Hs.617329 are likely to represent the same gene, which is one of the many versions of the human LINE-1 retrotransposon. Although the cDNA sequences from the two clusters are polyadenylated and do not overlap, they align very closely to each other on the human chromosome 4 p15.33 and, according to Blastp results, the deduced ORFs from each one match different regions of the ORF2 protein of LINE-1 retrotransposon element. Interestingly, we identified two other genes (Hs.570688 and Hs.385543) also matching domains of LINE-1 ORF2.

The putative promoter region of 22 genes had putative elements for 4 transcription factors, namely C/EBPalpha, NF1, Oct-1, and SP-1. Most had putative binding sites for another 11 transcription factors (Table S3).

### Experimental validation confirms highly restricted expression of three novel genes in melanoma or melanocyte lineage

**Figure 1:**
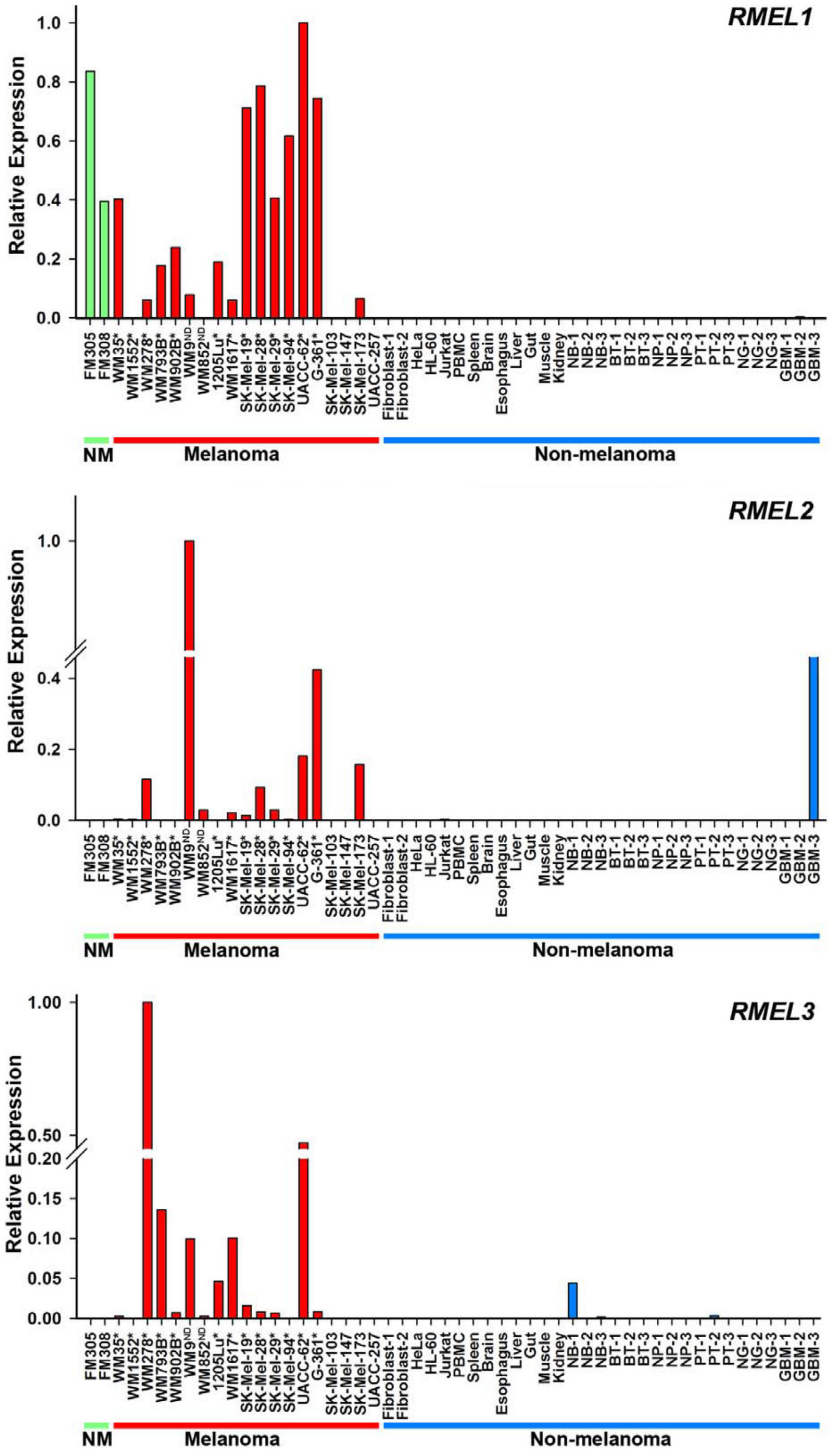
Validation by real-time RT-PCR of the melanoma/melanocyte-restricted expression of *RMEL1, 2* and *3*. Relative expression was calculated according to 2^−ΔΔCT^ method, using TBP (Tata-box binding protein) as endogenous control and the sample displaying the lowest normalized Ct as reference. The tissue panel included normal melanocytes (NM), melanoma cell lines (melanoma), including those harboring the activating BRAF V600E mutation (*), wild type BRAF (not marked), or with mutation status not determined (ND); and various cell lines and tissues of non-melanocytic origin (non-melanoma), including primary skin fibroblasts, HeLa, HL-60, Jurkat cells, peripheral blood mononuclear cells (PBMC), and necropsy samples from spleen, brain, esophagus, liver, intestine, skeletal muscle, kidney, normal bladder (NB), bladder tumor (BT), normal prostate (NP), prostate tumor (PT), normal glia (NG), gliobastoma (GBM).

For validation experiments we selected UniGene clusters composed of three or more GenBank sequences (Tables S2 and S4), excluding Hs.551051 and Hs.617329 that required further analysis to clarify whether they represent a single gene. Primer efficiency tests revealed six of nine genes with very low expression levels (Ct>34). Thus, we focused on the other three genes (Hs.295012, Hs.518390, Hs.559350) showing more consistent and reliable expression levels. As shown in Figure 1, expression analysis in a diverse panel of human cell lines and tissues confirmed highly restricted expression of these three genes in melanocyte/melanoma cells, so we named them as *RMEL1* (Hs.295012), *RMEL2* (Hs.518391), and *RMEL3* (Hs.559350). Except for very low levels found in a single glioblastoma sample (GBM-2), *RMEL1* mRNA expression was detected only in samples from melanocytic origin, including the two primary melanocyte cultures and 14 of 19 melanoma cell lines, confirming its highly restricted expression in melanocytic cells (Figure 1A). *RMEL2* mRNA expression was detected in 13 out of 19 melanoma cell lines, and no expression was detected in melanocytes, nor in 29 samples from other cell and tissue types (Figure 1B). However, despite the lack of expression in normal glia, high levels were detected in one (GMB-3) out of three glioblastoma samples (Figure 1B), suggesting that this gene, and perhaps *RMEL1* as well, are part of a common tumorigenic tract involved in these two types of neuroectoderm-derived tumors. *RMEL3* mRNA expression was detected in 13 out of 19 melanoma cell lines and in low levels in two normal bladder samples and one prostate tumor (Figure 1C). No expression was detected in melanocytes nor in 28 samples from other types of cells and tissues. Interestingly, there was a positive correlation between the expression of these three genes and the occurrence of oncogenic mutation of *BRAF* (Figure 1A–C).

**Figure 2:**
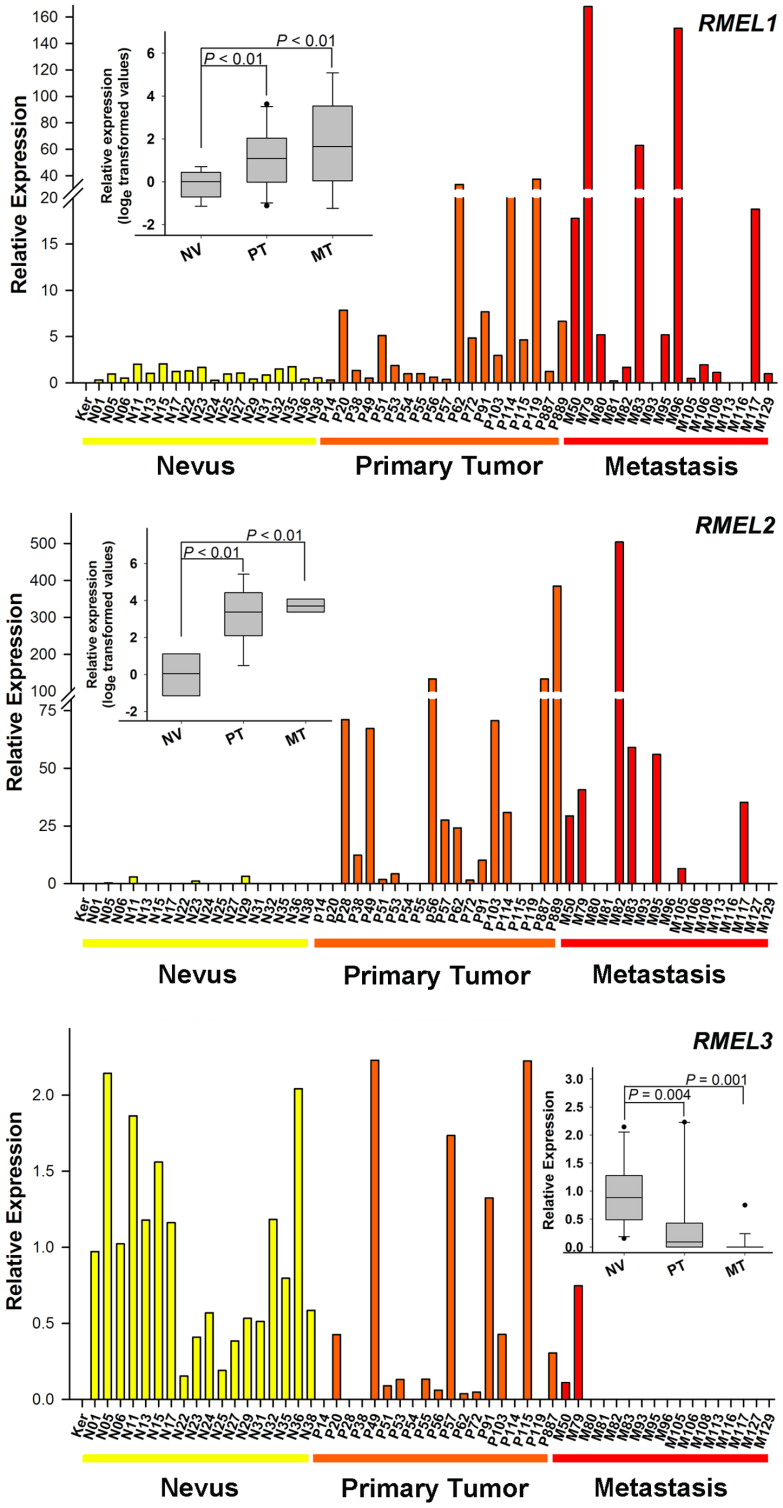
Expression profiles of *RMEL1, 2* and *3* during melanoma progression. Relative expression was calculated according to 2^−ΔΔCT^ method, using TBP (Tata-box binding protein) as endogenous control and the mean value of the normalized Cts of all nevi samples as reference. cDNA samples derived from keratynocytes (Ker), nevi (N), primary melanoma tumors (P) and melanoma metastasis (M), from different patients (distinguished with a number following the letter N, P or M) were analyzed by Real-Time PCR. Statistical analyses were performed using ANOVA (after log_e_ transformation of the data), for RMEL1 and RMEL2, or the Mann–Whitney test for RMEL3. Metastatic samples numbered 79, 81 and 105 are from lymph node metastasis; 50, 80, 93, 95, 96, 108, 113 and 127 from skin metastasis; 106 from lung metastasis and 82, 83, 116 and 129 from visceral metastasis.

We next analyzed the expression profile of these genes in a panel of nevi, primary, and metastatic melanoma tissues, including a sample from primary cultured keratinocytes as control (Figure 2). Real-time RT-PCR results showed lack of mRNA expression in keratinocytes, while significant differential expression was detected for the three genes in nevi and melanomas of both stages. Both *RMEL1* and *RMEL2* showed up-regulation in about 45 to 50% of the tumor samples. On the other hand, *RMEL3* exhibited the inverse expression pattern, marked by relatively high levels in nevi and progressive loss during melanoma progression, as revealed by its loss in 31% of the primary tumors and in 88% of the metastatic tumors (Figure 2A–C).

### Correlations of gene expression and clinical data provide insights into the role of RMEL genes in tumor progression

**Figure 3:**
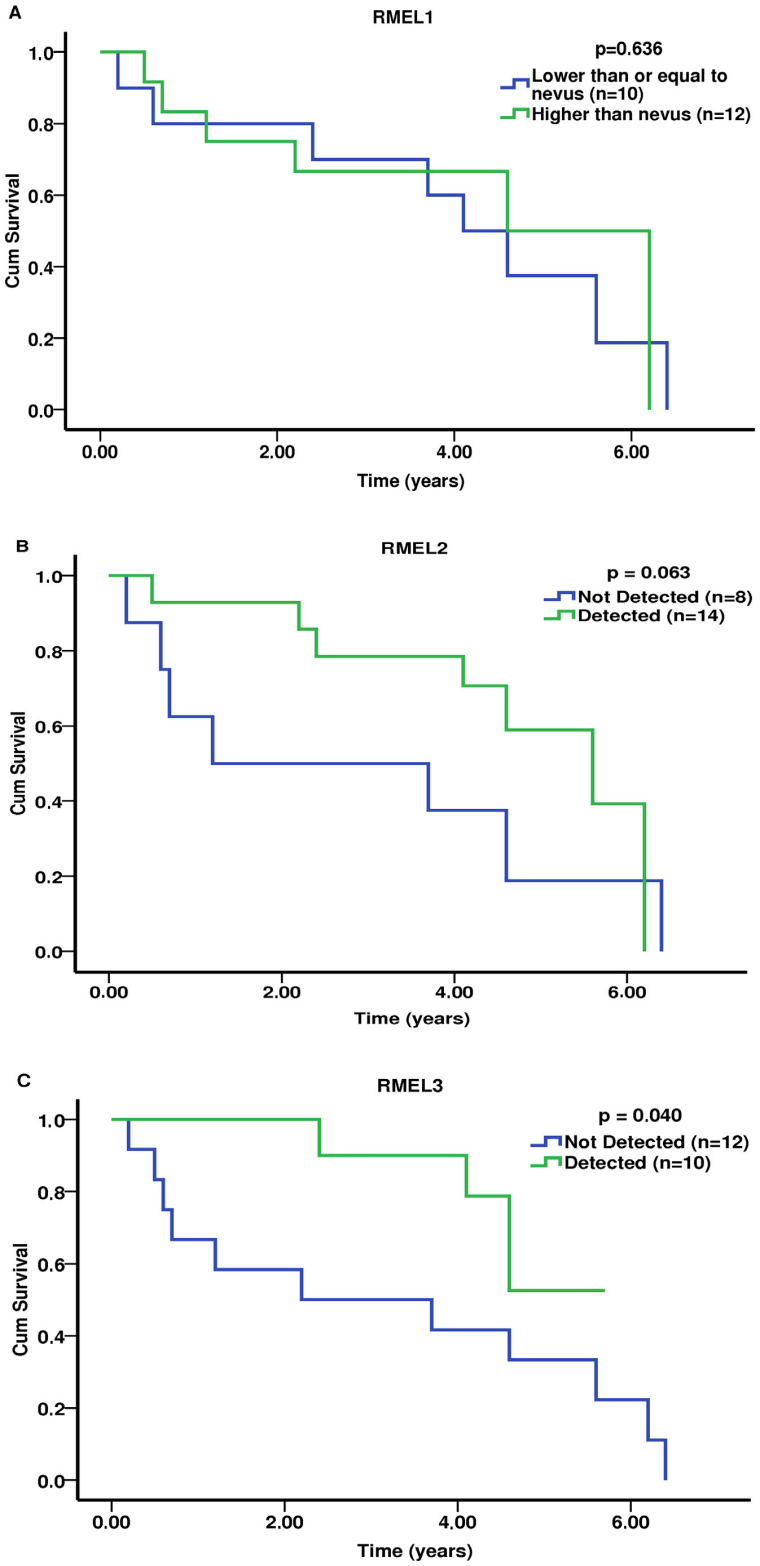
Kaplan-Meier estimates of cumulative survival for melanoma patients according to the expression of *RMEL1* (A), *RMEL2* (B) and *RMEL3* (C). Patients were divided into two groups according to expression levels, classified as lower or equal and higher than the maximal detected in nevi, for *RMEL1*, and as detected (D) and non-detected (ND), for *RMEL2* and *RMEL3*. The Kaplan-Meier survival curves were calculated using the SPSS Statistics version 18.0 from SPSS (IBM Company).

We performed a small scale study to correlate RMEL expression levels with clinical and histopathological features. Although we did not have data for all patients included in the expression study, we did so for a limited number of patients, allowing us to extract some correlations that provide initial clues of possible roles for the RMEL genes to guide future studies. We divided the patients into two groups according to the expression levels, smaller or equal and greater than the maximal expression observed in nevi, for RMEL1, and detected (D) and non-detected (ND), for RMEL2 and RMEL3. Expression levels of RMEL1 showed correlation with skin color (15 of 16 patients with expression of RMEL1 lower/equal than nevi were white, while only 9 of 14 were white in the group with high expression, p = 0.038) and tumor thickness (3/7 patients with low or lack of expression presented Clark levels IV/V, the other 4/7 were Clark I/II, in contrast 8/8 patients with high expression had Clark levels IV/V, p = 0.012). However, there was no correlation of RMEL1 expression with other relevant features of tumor progression or patient outcome (Figure 3A). RMEL2 expression correlated significantly (p = 0.026) with lymph node status, such that 73% (11/15) of the patients with detectable expression in the tumor showed the presence of tumor cells in lymph nodes against 33% (5/15) in the group lacking expression. Interestingly, mortality due to cancer in lymph node positive patients was 67% in the group with detectable expression of RMEL2 and 100% in the group lacking expression. Moreover, all lymph node-negative patients with expression of the gene lived (4/4) without developing disease while regardless of being lymph node negative most patients lacking expression of the gene died (83%, 5/6) due to cancer or developed the disease (1/6) over equivalent periods of follow up. Overall mortality in the population analyzed was around 53% in the lymph node-positive group and 33% in lymph node negative. Cells from tumors with preserved expression of RMEL2 showed either spindle (4/9) or epithelioid (5/9) shapes while those from tumors lacking expression were all epithelioid (6/6), p = 0.025. Additionally, RMEL2 expression showed a tendency of correlation with improved survival rate (Figure 3B). For RMEL3, we observed a significant (p = 0.002) inverse correlation of gene expression and tumor progression, so that the majority (13/19, 68%) of the patients lacking RMEL3 expression in their tumor sample presented with metastasis, most of them (9/13, 77%) in an aggressive (visceral) stage of the disease, whereas in contrast only a minority (3/13, 23%) of the patients with preserved RMEL3 expression were diagnosed with metastasis. Also, the only two metastatic samples with preserved RMEL3 expression were derived from less advanced, lymph nodal or cutaneous metastasis while all advanced metastases lacked expression (Figure 2C). Compatible with a protective role for RMEL3, we observed correlation of expression with the absence of lymph vascular invasion in the primary tumor site (10/11 patients), while in contrast only 1 of 4 patients lacking RMEL3 expression showed no lymph vascular invasion (p = 0.002). Additionally, RMEL3 expression positively correlates (p = 0.001) with improved patient status, as 6 of 10 patients in the group with detectable expression lived without the disease in contrast to none of 12 over the same period of follow up in the group lacking RMEL3 expression. Survival curves calculated using Kaplan-Meier method show that lack of RMEL3 expression in the tumor correlates with poor survival rates, with most deaths occurring within 2.2 years of follow up, an interval in which all ten patients with detectable expression were still alive (Figure 3C).

### Structure, conservation, and predicted products for the three novel melanoma-restricted genes

**Figure 4:**
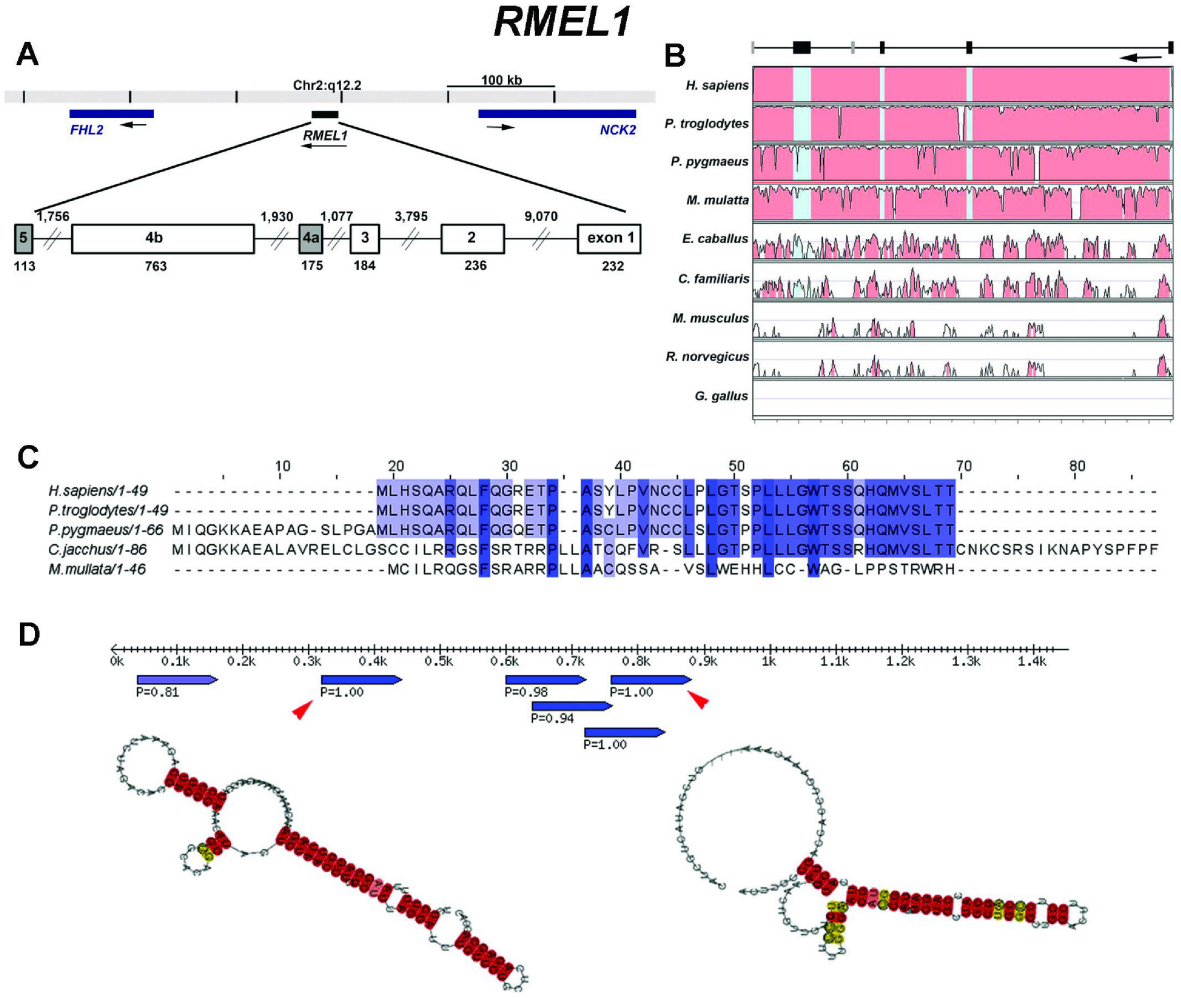
Structural organization, phylogenetic conservation, and predicted products for *RMEL1* (Hs.295012). (**A**) Genome context of the gene. The longest cDNA (BC038566) suggests a four-exon gene and ESTs (BE386026, BU156136 and BQ217207) suggest two more exons (shaded boxes). Numbers indicate intron and exon lengths in nucleotides. (**B**) VISTA plot displaying the conservation of the gene in nine species. The upper and lower limits of the box representing each species correspond, respectively, to 100 and 50% of sequence identity. (**C**) Multiple alignment of the deduced amino acid sequences of the *RMEL1* longest AUG-starting ORF of several primate species. (for all species, ORFs were deduced from putative transcribed sequences obtained by alignments of the human cDNA BC038566 against the genomic sequences). (**D**) Diagram of RNA secondary structures predicted by RNAz software. Rule indicates RNA length; pointed blue bars indicate segments predicted to form hairpin structures, and the numbers indicate the *RNA-class probability* (P), considered significant when greater than 0.5. Predicted RNA secondary structures for two of the segments (indicated by arrow heads) are shown.

**Figure 5 pone-0013510-g005:**
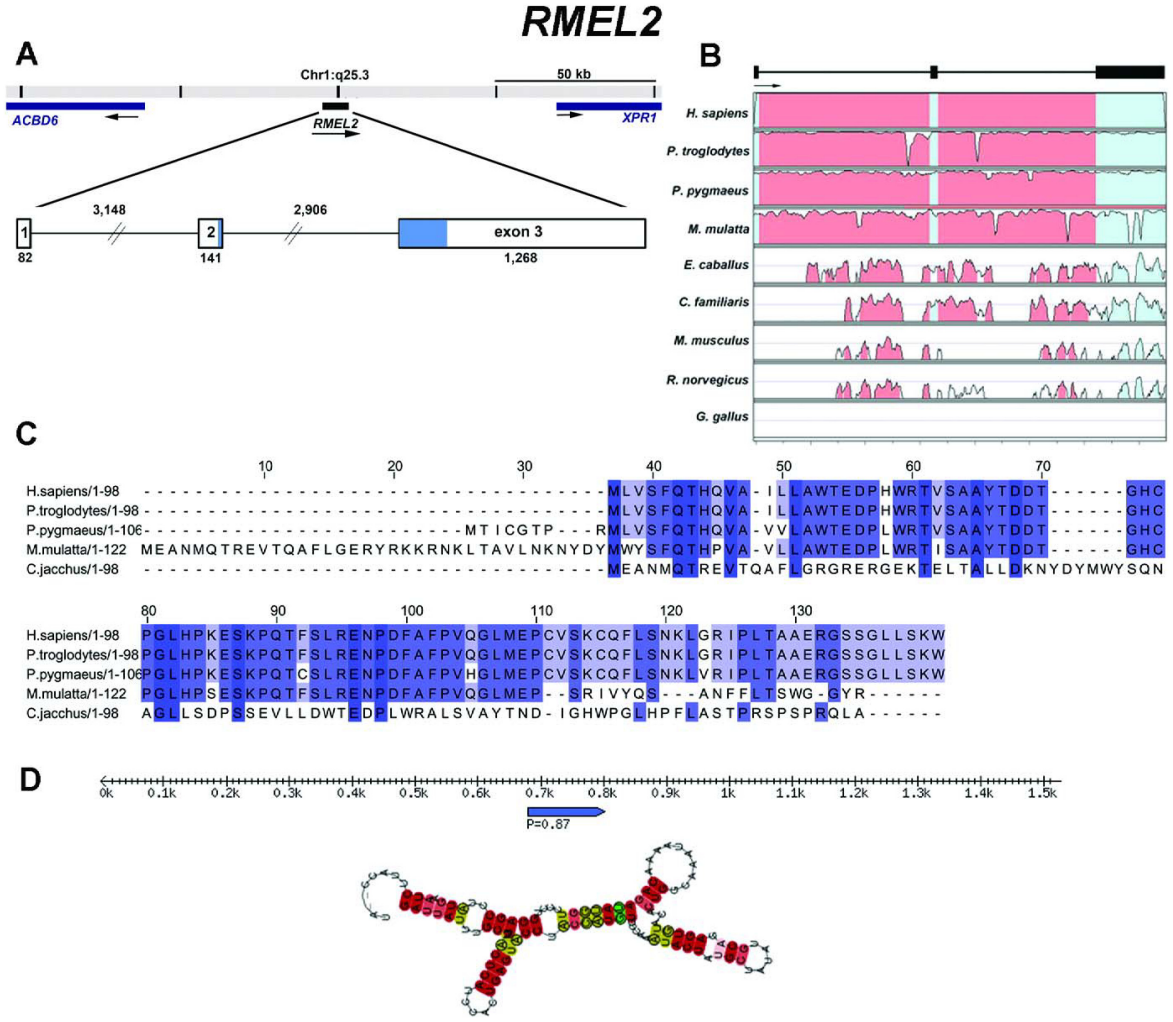
Structural organization, phylogenetic conservation, and predicted products for *RMEL2* (Hs.518391). (**A**) Genome context of the gene. The numbers indicate the intron and exon lengths in nucleotides. The region in blue represents the longest ORF found, spanning 294 bp and encoding 98 aa. (**B**) VISTA plot displaying the conservation of the gene in nine species. The upper and lower limits of the box representing each species correspond, respectively, to 100 and 50% of sequence identity. (**C**) Multiple alignment with ClustalW of the deduced amino acid sequences for the longest AUG-starting ORF of *RMEL2* in several primate species (for all species, ORFs were deduced from putative transcribed sequences obtained by alignments of the human cDNA BC063324 against the genomic sequences). (**D**) Diagram showing an RNA secondary structure predicted by RNAz software. Rule indicates RNA length; pointed blue bar indicates a segment predicted to form the hairpin structure shown, and the number is the *RNA-class probability* (P) of the prediction, considered significant when greater than 0.5.

**Figure 6:**
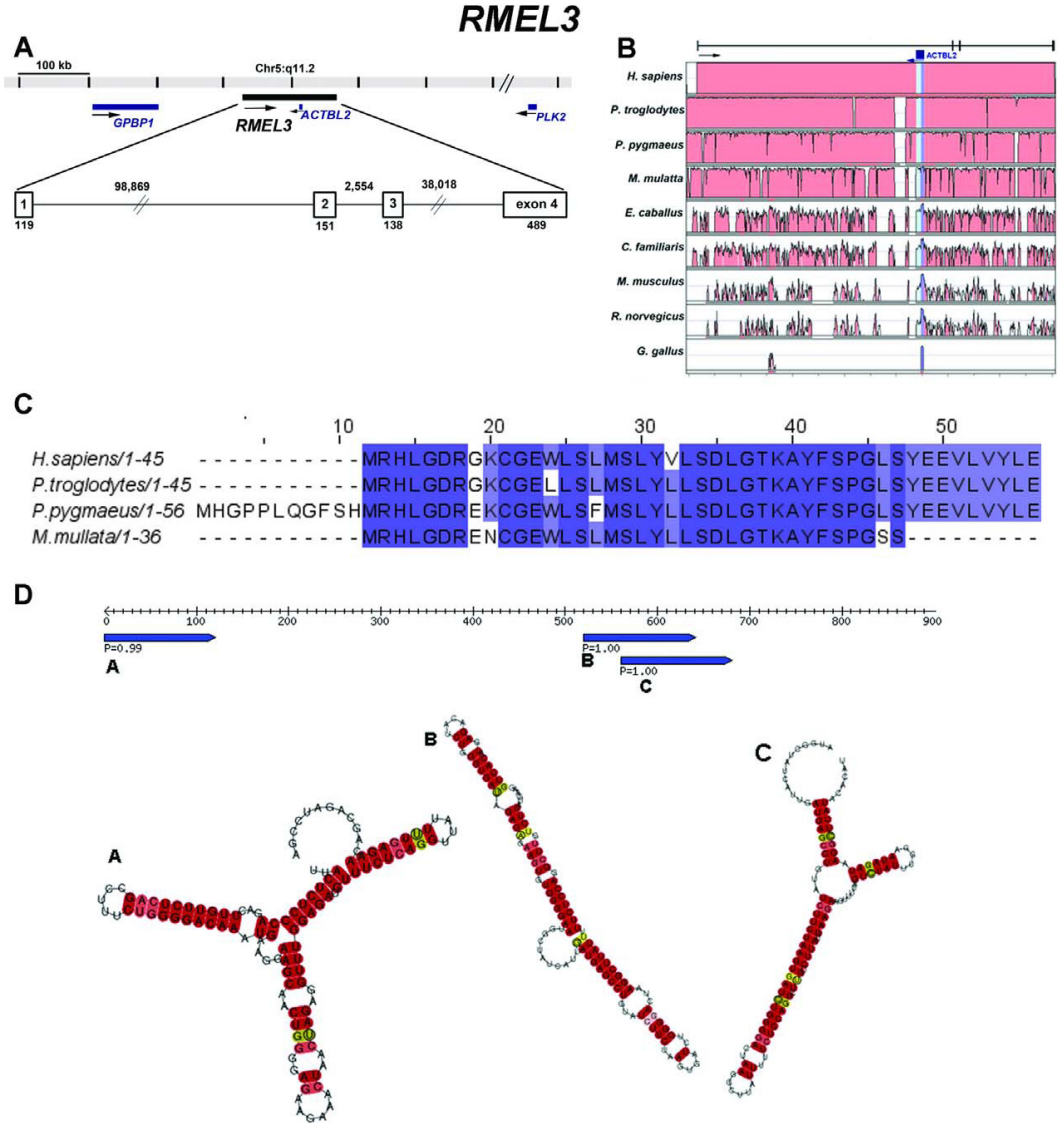
Structural organization, phylogenetic conservation, and predicted products for *RMEL3* (Hs.559350). (**A**) Genome context of the gene. ESTs available (BQ420825, CR746619 and BU161974) for the gene suggest the occurrence of four exons separated by large introns. In the opposite strand of the first intron there is an intron-less actin-like gene (*ACTLB2*). The numbers indicate the intron and exon lengths in nucleotides. (**B**) VISTA plot displaying the conservation of the gene in nine species. The upper and lower limits of the box representing each species correspond, respectively, to 100 and 50% of sequence identity. (**C**) Multiple alignment with ClustalW of the deduced amino acid sequences for the longest ORF, which is a primate-conserved AUG-starting ORF, in several primate species (for all species, ORFs were deduced from putative transcribed sequences obtained by alignments of the human EST BQ420825 against the genomic sequences). (**D**) Diagram of RNA secondary structures predicted by RNAz software. Rule indicates RNA length; pointed blue bars indicate segments predicted to form the hairpin structures shown, and the numbers indicate the *RNA-class probability* (P) of the prediction, considered significant when greater than 0.5.

The three validated genes are composed of multiple exons. *RMEL1* (Figure 4A) spans over a region of 19,212 bp on chromosome 2q12.2 and includes six exons supported by EST data, four of which are constant and two alternatively spliced. Although the UniGene cluster of this gene is composed of 16 expressed sequences, 1 derived from polyadenylated mRNA (long cDNA) and 15 ESTs, the UCSC Genome Browser shows the same sequences as the UniGene cluster plus 4 ESTs, totalizing 20 expressed sequences, derived from four different melanoma libraries, mapping to this locus. *RMEL2* spans 7,545 bp and is composed of three exons mapped to chromosome 1q25.3 with no evidence of alternative transcripts (Figure 5A). Also for this gene, the UCSC Genome Browser indicates the existence of additional expressed sequences (6 cDNAs and 11 ESTs) mapping to this locus in comparison to the UniGene cluster (12 ESTs). The third gene, *RMEL3*, maps to an extension of 138,365 bp on the chromosome region 5q11.2 and is organized in four exons separated by very large introns. There are only three ESTs representing this gene according to the UniGene and UCSC Genome Browser as well. Curiously, an intron-less gene annotated as *ACTBL2* maps to the longest intron of RMEL3 (Figure 6A).

All three genes show very low sequence conservation even within mammals. According to BLAT and VISTA alignments, the three complete genes are well conserved only throughout the various primate genomes (Figures 4-6, B). They are partially conserved in dog and horse, and only *RMEL2* has conserved portions of exonic sequences (exon 3) in mouse and rat (Figure 5B). The CPC software predicted that these genes have no coding potential, but *RMEL2* has been classified as a weak non-coding sequence. They have several stop codons in all possible frames, but some short ORFs can be detected in their sequences (Figures S1, S2, S3). We searched the sequences for the presence of ORFs with both the canonical AUG-start codon and alternative non-AUG codons, since there is an increasing number of reports showing the use of alternative translation initiation sites in mammals [15]. For *RMEL1*, the longest ORF, which is conserved across the various primates, is an AUG-starting ORF encoding a 49 aa long polypeptide (Figure 4C). However, assuming the use of alternative start codons, this gene has a longer ORF encoding a polypeptide of 91 aa residues. This is not a conserved ORF and it may have arisen recently from a single base pair alteration that creates a UGG codon for tryptophan, in place of the UGA-termination codon found in other primate genomes (Figure S4). For *RMEL2*, the longest putative ORF encodes a polypeptide chain of 98 aa residues, whose sequence is conserved within primates. Interestingly, the corresponding ORF in *M. mulatta* predicts a polypeptide with an additional 36 aa N-terminal (Figure 5C). For *RMEL3*, the longest putative ORF is an AUG-starting ORF that encodes a 45 aa long polypeptide conserved in the other three primates (Figures 6C and Figure S3).

We also considered the possibility that the genes could represent non-coding RNAs. We have not found any matches between these RNA sequences and previously characterized or predicted non-coding RNAs whose sequences are available in RNA databases. However, RNA secondary structures were predicted from their sequences, according to the RNA_Z_ software, which predicts RNA functional structure based on both, structural conservation and thermodynamic stability. For each RMEL gene, the RNA structure prediction was based on multiple alignments of five primate sequences (*Homo sapiens*, *Pan troglodytes*, *Pongo pygmaeus, Macaca mulatta* and *Callithrix jacchus*). The software provides an overall *RNA-class probability*. If this *probability* is >0.5 (the closer to 1.0 this value is, the more confident is the prediction), the alignment is classified as “functional RNA”, indicating that the sequence is from a potential RNA element in ncRNAs or a cis-regulatory element of mRNAs. For *RMEL1* (Figure 4C) and *RMEL3* (Figure 6C), five and three RNA structure predictions with very high probability (*RNA-class probability* >0.95) were generated, whereas for *RMEL2* (Figure 5D) there was only one prediction and with lower probability (*RNA-class probability*  = 0.87), compatible with the prediction that *RMEL2* is most likely a protein coding gene.

## Discussion

We developed a pipeline tool for EST data mining that is simple but effective in identifying genes displaying tissue-specific expression, according to our validation results. Most of the 38 UniGene clusters initially indicated by the analysis were indeed composed only of sequences derived from melanoma libraries and we validated the expression profile for three genes selected for experimental validation from the final list of 29 clusters, indicating that other candidates from the list should be validated as well in the future. Due to the high stringency of our search, ESTs for genes traditionally known as melanocyte/melanoma-specific genes, such as TYR (Hs.503555), DCT (Hs.301865), MLANA (Hs.154069), and SILV (Hs.95972), were not selected because they also match entries of other tissue datasets. Although we might have missed relevant genes with differential expression, we selected genes with highly specific expression. The use of such genes could improve the detection of early metastasis and help predict patient outcome. Since the expression of RMEL1 and RMEL2 is very specific to melanocyte/melanoma cells and their expression is preserved in great number of tumor samples, they are both valuable candidates, as molecular markers, for example to detect circulating tumor cells in the blood of melanoma patients. Given the correlation with clinical aspects, RMEL2 and RMEL3 expression might be valuable as prognostic markers and assessment of treatment response.

Most of the genes identified here seem to encode short peptides, and five out of the seven deduced proteins with characterized structural motifs contain nucleic acid-binding domains. Four of the deduced proteins match regions of the ORF2 of the retrotransposon element LINE-1. These elements, representing 17% of the human genome, are not frequently expressed in adult somatic cells, and evidence suggests that their transcripts may have oncogenic roles (for review [16]). It is also interesting to point out that almost 35% of the genes identified in this study have no predicted coding potential and might represent non-coding RNAs.

Of the three genes validated, *RMEL1* seems to be a novel melanocytic lineage-specific gene with significant up-regulation in primary and metastatic tumors. Whether it plays a role in melanocyte differentiation and melanoma progression, like other melanocyte-specific genes such as *MITF* [17], [18], is an interesting matter for future studies. Of note, *RMEL1* expression was far more restricted to melanocytic cells than that observed for canonical melanocyte-specific genes.

RMEL2 was the gene most strictly associated to malignancy in melanoma and possibly glioblastoma, since it is not expressed in normal melanocytes or glia and is barely detected in nevi, yet it is up-regulated in ∼50% of the melanomas (and 1 of 3 glioblastomas). RMEL2 maps to 1q25.3, a region (1q23.3–q25.3) frequently amplified in melanomas [19]. Therefore, it is possible that *RMEL2* is up-regulated in melanoma by means of genomic amplification. Future functional studies will be required to discern its disease significance from merely a bystander in the process.

*RMEL3* shows an intriguing expression profile, suggestive of a possible role for this gene in nevi and as a tumor or metastasis suppressor. Although melanocytic nevi are benign lesions that rarely progress to malignant melanoma, they are altered in relation to normal melanocytes and frequently exhibit the oncogenic activating *BRAF* mutation V600E [20], [21]. It has been proposed that nevi cells suffer an initial proliferation step, and are growth arrested by an oncogene-induced senescence mechanism [22]. Recent work identified several genes required for the BRAFV600E-induced senescence and showed that in the context of BRAF mutation, expression loss of one of them, *IGFBP7*, is critical in promoting the progression to melanoma [23]. *RMEL3* profile resembles that of *IGFBP7* in some ways, since its expression is not detected in melanocytes while it is up-regulated in nevi and absent from most metastases and about half of the primary tumors. Also, mapping of the conserved gene *ACTBL2* to an *RMEL3* intron may represent a meaningful regulatory interrelationship with reflex in the actin cytoskeleton dynamics and cell migration. Future research to uncover the function of this gene may reveal novel insights into the molecular alterations of melanocytic nevi implicated in melanoma progression.

Curiously, the three RMEL genes were predicted as having no coding potential. Other genes previously classified as non-coding RNA genes have been shown to encode short functional peptides [24], [25]. Of the three genes analyzed, *RMEL2* has the highest probability to encode a protein, and although the other two had AUG-starting ORFs encoding fewer than 50 amino acid residues, a longer ORF starting at an alternative codon was detected for *RMEL*1. This ORF (encoding 91 aa) is present only in the human sequence and may have originated from an ancestral non-coding sequence, as recently proposed in an interesting work that provides evidence for the origin of three novel human-specific ORFs from ancestral non-coding sequences [26]. Besides the possibility of these genes encoding polypeptides that are primate- or even human-specific, they are also candidates for encoding primate-specific functional non-coding RNAs. However, more conclusive evidence regarding evolutionary conservation of these genes will only be possible after the actual encoded products are defined.

Large scale studies have demonstrated that most regions of eukaryotic genomes are transcribed as non-coding RNAs and an increasing number of these molecules have been implicated in many regulatory processes [27], [28]. Notably, several non-coding regulatory RNAs, microRNAs (miRNAs), were recently discovered as involved in critical steps of the tumorigenesis and metastasis cascades, in several types of cancer including melanoma [29], [30], [31]. According to the miRBase, there are no annotated or predicted miRNAs mapping to the RMEL genomic regions. Thus, one possibility is that RMELs encode miRNAs not previously identified due to their highly restricted tissue expression pattern. Another possibility is that they belong to the class of long non-coding RNAs. These types of RNAs are very heterogeneous, and novel long non-coding RNAs are difficult to identify based on sequence analysis because they are not very well conserved throughout the phylogenetic scale [28], [32]. Indeed, the three genes identified here are significantly conserved throughout primate genomes only. Interestingly, besides miRNAs, several short and long non-coding RNAs have also been implicated in cancer development and described as cancer markers [28]. An interesting example is DD3/PCA3, a prostate cancer-specific RNA showing low conservation in non-primate mammals [33], [34]. Although no role for this gene in prostate cancer has been proposed, detection of DD3 RNA in body fluids proved useful for predicting prognosis [35], [36]. No studies concerning diagnostic applications of non-coding RNAs are available for melanoma. although there is great expectation towards this end (see[31]).

In summary, we here presented a new strategy for extracting valuable information from expressed sequence databases, and generated a list of 28 potential genes, represented by 29 UniGene clusters, with validated probability of being involved in melanoma. To our knowledge, the three validated genes shown here were found to have the most restricted expression patterns ever seen for melanoma and we presume that future functional studies of these genes will shed new light on melanocyte and melanoma biology. The small scale correlative study of gene expression and clinical data shown here lead us to suggest that especially RMEL3 is likely to play an active role as a tumor and metastasis suppressor, but also RMEL2 may function to impose constraints to the initial development of the primary tumor and perhaps has a role in invasion. Thus, we suggest that future investigation will be worthwhile to assess the relevance of RMEL2 expression as a marker to predict disease outcome in lymph node-negative patients and RMEL3 as a general marker of melanoma patient outcome. For RMEL1 we suggest a possible involvement in differentiation/pigmentation since it is expressed in normal melanocytes and this might explain some correlation with skin color in melanoma patients. Concluding, we expect that the data presented here offers promising new targets for future validation in large clinical studies as diagnosis markers or for development of therapeutic strategies.

## Materials and Methods

### Software development and implementation

Using Perl scripts all the reports of the dbEST database were downloaded and locally inserted into a MySQL database. All ESTs corresponding to *Homo sapiens* were selected and then, a human tissue source list were created collecting all the terms added to six fields (Cell line, Cell type, Description, Lab name, Tissue type and Title) of the dbEST submission files (606 terms). The human UniGene reports were also downloaded as text files and then were pre-processed to eliminate unnecessary fields; the remaining fields were indexed, using ‘MySQL’. The process of identification of tissue-specific genes starts by selecting a keyword from the list of tissue source terms. The selected term is the input argument for the search engine. The MySQL Database Management System separates the ESTs in two groups: a) ESTs whose tissue source matches the selected keyword and b) ESTs whose tissue source does not match the selected keyword. After the separation, each EST is associated to its corresponding UniGene cluster. Then, the search engine compare the two lists of clusters generated in the first step in order to identify clusters formed only by ESTs belonging to libraries matching the keyword. Based on these criteria we detect only the ESTs that are tissue-specific.

The implementation of the methodology described above was done using Perl scripts, which basically trigger SQL commands to the MySQL database, to perform the searches. The results of the SQL commands are then stored by the Perl script for further comparison of clusters lists. The results found by the Perl script are then sent to a PHP script, for correct formatting and HTML presentation. This PHP script is also responsible for controlling the HTML form that gets the selected keyword and sends it to the Perl script. All the developed scripts and the MySQL database are running into a GNU/Linux server. The tool named EST2TSE (EST to Tissue Specific Expression) is available at http://www.prometheus.fmrp.usp.br/EST2TSE.

### Selection of melanoma-specific genes (EST clusters)

Using the methodology described above and the dbEST [37] and the Unigene data as of January 2007, we were able to separate the EST reports into two groups: (i) ESTs whose tissue origin was melanoma (Melanoma group: 129,906 sequences); and (ii) ESTs derived from any tissue except melanoma (Non-melanoma group: 7,895,596 sequences). Melanoma sequences were derived from 12 melanoma libraries, the largest one containing 41,936 sequences and the smallest 19 sequences. We next associated the melanoma and non-melanoma ESTs to UniGene clusters and compared the two lists of clusters in order to identify clusters formed by ESTs found in the melanoma group only.

### Cell culture

The following human cells were used: primary foreskin melanocytes (FM305 and FM308); melanoma cell lines harboring the BRAF V600E/I mutation (*), wild type for this allele or with mutation status not determined (ND); RGP (radial growth phase) lines WM35*and WM1552*; VGP (vertical growth phase) lines WM278*, WM793*, and WM902*; metastatic lines WM9 (ND), WM852 (ND), 1205Lu*, WM1617*, SK-Mel-19*, SK-Mel-28*, SK-Mel-29*, SK-Mel-94*, UACC-62*, SK-Mel-103, SK-Mel-147, SK-Mel-173, and UACC-257; cervical carcinoma HeLa cells; Jurkat T cell leukemia; HL-60 promyelocytic leukemia cells; and primary skin fibroblasts.

WM melanoma cell lines were maintained as previously described [38]. All the other melanoma cells, the HeLa cells, and the primary cultured fibroblasts were maintained in Dulbeco's modified Eagle's medium (DMEM) supplemented with 10% fetal bovine serum (FBS), 50 U/mL penicillin, and 50 µg/mL streptomycin. The normal melanocytes were isolated from neonatal foreskin and maintained as previously described [39]. Jurkat and HL-60 were cultured in RPMI medium supplemented with 10% FBS, 50 U/mL penicillin, and 50 µg/mL streptomycin. We periodically monitored cell morphology and observed no changes in the cell lines. All cells were frequently checked for mycoplasm contamination by DAPI staining.

### Ethics Statement

The study protocol for the collection of all non-melanoma tumor and normal tissue samples was approved by the Ethics Committee of the Hospital das Clínicas da Faculdade de Medicina de Ribeirão Preto, Universidade de São Paulo (# 7645/1999 and 7645/2005), and written informed consent was obtained from each patient. Nevi and melanoma biopsy samples were obtained from patients at the Hospital do Câncer AC Camargo, São Paulo, with written informed consent provided by all patients, and according to protocols approved by the Ethics Committee of the Hospital do Câncer AC Camargo (# 560/03 and 567/04).

### Processing of the biopsy and necropsy samples

The non-melanoma samples were processed as previously described [40]. In the case of the nevi and melanoma samples, after biopsy, all of them were split in two fragments: one was maintained in RNAlater (Ambion, Austin, TX) and the second was processed for hematoxylin-eosin staining and pathologic analysis. Fragments kept on RNAlater were processed when histopathological diagnosis was confirmed. All tissue fragments were microdissected for maximal exclusion of non-melanocytic cells.

### RNA extraction and real time RT-PCR

Total RNA was isolated from all human cell lines, non-melanoma biopsy, and necropsy samples using TRIzol (Invitrogen, Carlsbad, CA) according to manufacturer's instructions. RNA integrity was checked by formaldehyde-agarose gel electrophoresis for all samples and then 1 µg of DNase-treated RNA (DNA-free kit, Ambion, Austin,TX) was converted into cDNA using the High-Capacity cDNA Reverse Transcription kit (Applied Biosystems, Foster City, CA).

Total RNA from nevi and melanoma biopsies was isolated using the RNeasy Midi kit (Qiagen, Hilden, Germany) and then submitted to a mRNA amplification as protocol previously described [41], with modifications. Briefly, 3 µg of total RNA were used for first and subsequent second strand cDNA synthesis using the ImProm-II reverse transcription system (Promega, Madison, WI). Ds-cDNA was cleaned up by phenol/chlorophorm/isoamyl alcohol extraction and ammonium acetate precipitation. Amplified RNA (aRNA) was obtained by *in vitro* transcription from 10 µl of ds-cDNA using the RiboMAX system (Promega, Madison, WI) and subsequent purification using TRIzol (Invitrogen, Carlsbad, CA). For the final cDNA synthesis, 1 µg of aRNA was converted to cDNA using the RT Improm II system (Promega, Madison, WI).

Equal amounts of each cDNA were analyzed by real-time PCR using specific primers (400 nM) and SYBR Green PCR Power Mix (Applied Biosystems, Foster City, CA) in an ABI PRISM 7500 Sequence Detection System (Applied Biosystems, Foster City, CA). Each sample was measured in duplicate. All primer sequences are listed in Table S4. To avoid amplification of undesirable genomic DNA, we designed primer pairs flanking introns. All primers were submitted to efficiency tests using serial dilutions of mixture of cDNAs from five melanoma cell lines (WM35, WM793, WM9, 1205, and WM1617). Only primer pairs presenting efficiency in the range of 90–110% were used. Cycle threshold (Ct) was converted to relative expression according to the 2^−ΔΔCT^ method, using TBP (TATA-box binding protein) as endogenous control. For data analysis, in the case of cell lines and non-melanoma tissues, sample displaying the lowest normalized Ct was taken as reference, and in the case of melanoma tissues, reference was the mean value of the normalized Cts of all nevi samples. Statistic significance (*P*<0.05) of the differences in the expression levels was determined using ANOVA (SAS software, version 9.0), after log_e_ transformation of the data, for the genes RMEL1 and RMEL2, or the Mann–Whitney test (SigmaPlot software) for RMEL3.

### Statistical analysis

Statistical significance (*P*<0.05) of the differences in the expression levels of RMEL genes among the sample groups (nevi, primary tumors and metastasis) was determined using ANOVA (SAS software, version 9.0), after log_e_ transformation of the data, for the genes RMEL1 and RMEL2, or the Mann–Whitney test (SigmaPlot software) for RMEL3. The correlations between the gene expression and clinicopathologic variables were evaluated by the Likelihood Ratio Chi-square test, using the software JMP version 6.0.0 from SAS Institute Inc. Patient survival curves were calculated by the Kaplan-Meier method, with the statistical significance evaluated by the Breslow (Generalized Wilcoxon) test, using the SPSS Statistics version 18.0 from SPSS (IBM Company).

### Sequence analysis

For analyses of coding potential and definition of the deduced ORFs we used the software Coding Potential Calculator (CPC) [42] and the software Translator [43]. We used default parameters to perform the analyses and accepted the CPC pre-defined decision scores. We used the Translation Map tool [44] for translation maps. Before the ORF analyses, all human cDNA sequences were aligned to the human genome and those presenting sequence mismatches were corrected on the basis of the genomic sequence. Human genomic sequences of interest, obtained from the UCSC Genome Browser [45], were aligned to other genomes using the GenomeVISTA tool [46], generating a phylogenetic conservation plot. RNA secondary structure of *RMEL1, RMEL2*, and *RMEL3* was predicted using the RNAz software [47], [48]. The putative cDNA sequence of each gene of interest from four primate species was deduced by alignments with the human cDNA sequences (*RMEL1*: BC038566, *RMEL2*: BC063624 and *RMEL3*: BQ420825) using the BLAT tool (UCSC Genome Browser). The sequences were then provided to Clustal W and the multiple alignments generated were used as input for the RNAz software. Putative transcription factor binding sites in gene promoters were predicted using the AliBaBa 2.1 tool [49] that uses the TF (transcription factor) binding site database TRANSFAC. Putative promoter regions of all genes represented by cDNA sequences longer than 700 bp were included in our analysis. Genomic regions mapping at 2.0 kb upstream of the cDNA sequence were obtained with the UCSC GenomeBrowser and submitted to AliBaBa 2.1, generating a list of TF binding sites for each promoter. Using a locally developed script, all lists were compared to each other and a single list displaying the frequency of all TF elements detected for each promoter was generated.

## Supporting Information

Table S1 Comparison of EST2TSE with other tools for tissue specific gene selection from EST databank.
(0.03 MB DOC)

Table S2 UniGene clusters containing ESTs detected exclusively in melanoma libraries.
(0.06 MB DOC)

Table S3 Most frequent transcription factor binding sites in promoter regions of the genes represented by ESTs found exclusively in melanoma.
(0.03 MB DOC)

Table S4 Primers used for amplification by Real Time RT-PCR.
(0.04 MB DOC)

Figure S1 Putative ORFs and deduced amino acid sequences for *RMEL1*.
(0.08 MB DOC)

Figure S2 Putative ORFs and deduced polypeptide sequences for *RMEL2*.
(0.09 MB DOC)

Figure S3 Putative ORFs and deduced amino acid sequences for *RMEL3*.
(0.06 MB DOC)

Figure S4 A single base pair mutation creates a putative human lineage-specific ORF in *RMEL1* gene.
(0.14 MB DOC)
