# Supplementary material for: Novel Primate-Specific Genes, RMEL 1, 2 and 3, with Highly Restricted Expression in Melanoma, Assessed by New Data Mining Tool
Source: PLoS One. 2010 Oct 20;5(10):e13510. doi: 10.1371/journal.pone.0013510 (PMC2958148; doi:10.1371/journal.pone.0013510)
Supplement: Table S1 — Comparison of EST2TSE with other tools for tissue specific gene selection from EST databank. (0.03 MB DOC) [file pone.0013510.s001.doc]

| **TOOL** | **SEARCH POSSIBILITIES** | **DETECTION METHOD** | **USABILITY** | **URL** |
| --- | --- | --- | --- | --- |
| **EST2TSE** | Any tissue/condition in dbEST | Unique genes | Direct selection of tissues/condition | http://www.prometheus.fmrp.usp.br/EST2TSE |
| **GLS/CGAP** | Human/mouse tissues - normal and cancer conditions ( limited list of terms) | Unique/Non-unique genes | Direct selection of tissues/condition (limited list of terms) | http://cgap.nci.nih.gov/Tissues/LibrarySummarizer |
| **TiGER** | Human tissues (without distinction between normal and cancer) | Gene Expression Enrichment | Direct selection of tissues/condition | http://bioinfo.wilmer.jhu.edu/tiger/ |
| **UniGene/DDD** | Any tissue/condition in dbEST | Gene Expression Enrichment | Manual selection of libraries to compose the groups for each search | http://www.ncbi.nlm.nih.gov/UniGene/help.cgi?item=ddd |

**Table 1.** Comparison of EST2TSE with other tools for tissue specific gene selection from EST databank.
