# Supplementary material for: Novel Primate-Specific Genes, RMEL 1, 2 and 3, with Highly Restricted Expression in Melanoma, Assessed by New Data Mining Tool
Source: PLoS One. 2010 Oct 20;5(10):e13510. doi: 10.1371/journal.pone.0013510 (PMC2958148; doi:10.1371/journal.pone.0013510)
Supplement: Table S2 — UniGene clusters containing ESTs detected exclusively in melanoma libraries. (0.06 MB DOC) [file pone.0013510.s002.doc]

**Table S2: UniGene clusters containing ESTs detected exclusively in melanoma libraries**

| **Unigene ID** | **Description** | **Gene Symbol** | **Cluster size** | **Coding potential ^a^** | **Putative domains** | **Chrom. region** |
| --- | --- | --- | --- | --- | --- | --- |
| **Hs.166198** |  |  |  |  |  |  |
| **Hs.295012** | Hypothetical protein LOC285000 | **RMEL1** | 16 | No |  | Chr2(q12.2) |
| **Hs.518391** | Hypothetical protein LOC148756 | **RMEL2** | 12 | No (weak) |  | Chr 1 (q25.3) |
| **Hs.166198** | Synovial sarcoma, X breakpoint 5 | SSX5 | 8 | Yes (188 aa)^&^ | KRAB box (Kruppel - associated box) | chrX(p11.23) |
| **Hs.382776** | Hepatoma associated protein | HTA | 6 | Yes (92 aa)^&^ |  | Chr 16 (q22.3) |
| **Hs.551051** | clone IMAGE:3917723, mRNA |  | 6 | Yes (77 aa) | DUF1725 (ORF2,LINE-1) | Chr 4 (p15.33) |
| **Hs.617329** | Similar to hypothetical protein (L1H 3 region) - human, mRNA (cDNA clone MGC:46671 IMAGE:5563071) |  | 4 | Yes | Reverse transcriptase (RNA-dependent DNA polymerase domain ORF2,LINE-1) | Chr 4(p15.33) |
| **Hs.434302** | Hypothetical protein LOC340357 | LOC340357 | 4 | No (weak) |  | Chr 8 (p23.1-p22) |
| **Hs.632060** | Transcribed locus, strongly similar to XP_001136156.1(Pan troglodytes) |  | 4# | Yes (weak-68 aa) |  | Chr 8 (q21.3) |
| **Hs.351544** | Tubulin, beta polypeptide 4, member Q (TUBB4Q) | TUBB4Q | 3 | Yes (432 aa)^&^ | Tubulin/FtsZ family, GTPase domain | Chr4 (q35.2) |
| **Hs.551009** | Chromosome 21 open reading frame 117 | C21orf117 | 3 | No |  | Chr 21 (q21.1) |
| **Hs.559350** | Transcribed locus | **RMEL3** | 3# | No |  | Chr 5 (q11.2) |
| **Hs.570688** | clone IMAGE:2905386, mRNA |  | 3 | Yes (189 aa)* | DUF1725 (ORF2, LINE-1) | Chr 3 (q26.1) |
| **Hs.586239** | clone IMAGE:5111956, mRNA |  | 3 | No |  | Chr 6 (q14.1) |
| **Hs.652228** | Zinc finger protein 705D ZNF705D | ZNF705D | 3 | Yes (300 aa)^&^ | KRAB box e Zinc-finger C2H2 type domain | Chr 8 (p23.1) |
| **Hs.382317** | CDNA clone IMAGE:3922312 |  | 2 | No |  | Chr 9(q33.1) |
| **Hs.385543** | Transcribed locus, weakly similar to XP_001131241.1(Homo sapiens) |  | 2 | Yes (256 aa) | Endo-Exonuclease/ phosphatase (ORF2, LINE-1) | Chr 15 (q14) |
| **Hs.385790** | Chromosome 9 open reading frame 107 | C9orf107 | 2 | Yes (73aa) |  | Chr 9 (q31.1) |
| **Hs.406479** | clone IMAGE:5528009, mRNA |  | 2 | Yes (40 aa) |  | Chr 4(q32.3) |
| **Hs.407538** | clone IMAGE:5538960, mRNA |  | 2 | No |  | Chr 9 (q31.2) |
| **Hs.482753** | Transcribed locus, strongly similar to XP_001148198.1 hypothetical protein [Pan troglodytes] |  | 2# | Yes (120 aa) |  | Chr 1 (p35.2) |
| **Hs.497583** | Transcribed locus |  | 2# | Yes (97 aa) |  | Chr 14 (q32.2) |
| **Hs.547548** | clone IMAGE:5538612, mRNA |  | 2 | No |  | Chr 2 (p12) |
| **Hs.553957** | CDNA clone IMAGE:4623222 |  | 2 | Yes (49aa) |  | Chr 5 (q23.1) |
| **Hs.566753** | clone IMAGE:5541085, mRNA |  | 2 | No (weak) |  | Chr 7 (p14.1) |
| **Hs.568703** | Carbonic anhydrase XIV (CA14) pseudogene, mRNA (cDNA clone MGC:20106 IMAGE:4643256) | CA14 | 2 | Yes (50 aa)^&^ |  | Chr 1 (q24.1) |
| **Hs.570414** | CDNA clone IMAGE:5541115 |  | 2 | Yes (weak-115aa) | Transmembrane region | Chr 21 (q21.3) |
| **Hs.637822** | clone IMAGE:5548255, mRNA |  | 2 | Yes (weak-100aa) |  | Chr 1 (q25.3) |
| **Hs.650135** | Transcribed locus, moderately similar to XP_001515524.1 [Ornithorhynchus anatinus] |  | 2# | Yes (126aa)* | Helix-loop-helix DNA binding domain | Chr 2 (q36.1) |
| **Hs.651952** | CDNA clone IMAGE:6050961 |  | 2 | Yes (weak-59 aa) |  | Chr 18 (p11.21) |

a: coding potential predicted by CPC software.

&: deduced ORF from GenBank sequence report

# cluster containing only ESTs.

* Deduced ORF from ESTs whose sequence was corrected according genome alignment.
