# Supplementary material for: Novel Primate-Specific Genes, RMEL 1, 2 and 3, with Highly Restricted Expression in Melanoma, Assessed by New Data Mining Tool
Source: PLoS One. 2010 Oct 20;5(10):e13510. doi: 10.1371/journal.pone.0013510 (PMC2958148; doi:10.1371/journal.pone.0013510)
Supplement: Table S3 — Most frequent transcription factor binding sites in promoter regions of the genes represented by ESTs found exclusively in melanoma. (0.03 MB DOC) [file pone.0013510.s003.doc]

**Table S3: Most frequent transcription factor binding sites in promoter regions of the genes represented by ESTs found exclusively in melanoma.**

| **TFs** | **Frequency** |
| --- | --- |
| **C/EBPalpha** | **22/22^a^** |
| **NF-1** | **22/22** |
| **Oct-1** | **22/22** |
| **SP-1** | **22/22** |
| **GATA-1** | **18/22** |
| **AP-1** | **16/22** |
| **GR** | **16/22** |
| **NF-kappaB** | **16/22** |
| **SRF** | **15/22** |
| **TBP** | **15/22** |
| **C/EBPbeta** | **14/22** |
| **C-jun** | **13/22** |
| **ICSBP** | **13/22** |
| **HNF-1** | **12/22** |
| **HNF-3** | **12/22** |
