# Supplementary material for: Novel Primate-Specific Genes, RMEL 1, 2 and 3, with Highly Restricted Expression in Melanoma, Assessed by New Data Mining Tool
Source: PLoS One. 2010 Oct 20;5(10):e13510. doi: 10.1371/journal.pone.0013510 (PMC2958148; doi:10.1371/journal.pone.0013510)
Supplement: Table S4 — Primers used for amplification by Real Time RT-PCR. (0.04 MB DOC) [file pone.0013510.s004.doc]

**Table S4**. Primers used for amplification by Real Time RT-PCR

| Unigene cluster (cDNA/EST accession number) | Primer sequence |
| --- | --- |
| Hs.166198 (NM_021015) | F: 5’-CCTCAGGAAAACTAAATACCTC-3’ |
|  | R: 5’-CTCTTCATAAATCACCAGTTGC-3’ |
| Hs.295012 (BC038566) | F: 5’- GACTGGTTCTGGCTGGTTTG-3’ |
|  | R: 5’- GGGAACACACGCACAGTTTC-3’ |
| Hs.351544 (NM_020040) | F: 5’-CAGGCCAGACAACTTCATTTC-3’ |
|  | R: 5’-ACAACGTCCATCACTGACTCC-3’ |
| Hs.434302 (BC039487) | F: 5’-GACTGAAATTCCAACTCACTG-3’ |
|  | R: 5’-ATCACCTATGTAGGTCTGGAG-3 |
| Hs.518391 (BC063624) | F: 5’-AAGACTGAACTGACTGCTGTTC-3’ |
|  | R: 5’-GCCACTTGATGGGTCTGAAAG-3’ |
| Hs.551009 (BC009878) | F: 5’-CACTGAACTACCAATGCCTTTG-3’ |
|  | R: 5’-TCCCATCATTGAGGACAGGTG-3’ |
| Hs.559350 (BQ420825) | F: 5’-ATGTGCTCCAAGAAAACCAGAG-3’ |
|  | R: 5’-CTTTGTCACAGGAATACCCAAC-3’ |
| Hs.570688 (BC019327) | F: 5’-CTAATGGAATTGCACCACACC-3’ |
|  | F: 5’-AAGGCAGTGGAAATGAGCAAG-3 |
| Hs.632060 (BM555937) | R: 5’-ATTCTGAGACAGCAGGAAGAG-3’ |
|  | F:5’-GAGCTGTGATGTGAAGTTTCC-3’ |
| TBP (endogenous control) | F: 5’- AGCTGTGATGTGAAGTTTCC-3’ |
|  | R: 5’ TCTGGGTTTGATCATTCTGTAG-3’ |
