# Supplementary material for: Novel Primate-Specific Genes, RMEL 1, 2 and 3, with Highly Restricted Expression in Melanoma, Assessed by New Data Mining Tool
Source: PLoS One. 2010 Oct 20;5(10):e13510. doi: 10.1371/journal.pone.0013510 (PMC2958148; doi:10.1371/journal.pone.0013510)
Supplement: Figure S2 — Putative ORFs and deduced polypeptide sequences for RMEL2. (0.09 MB DOC) [file pone.0013510.s006.doc]

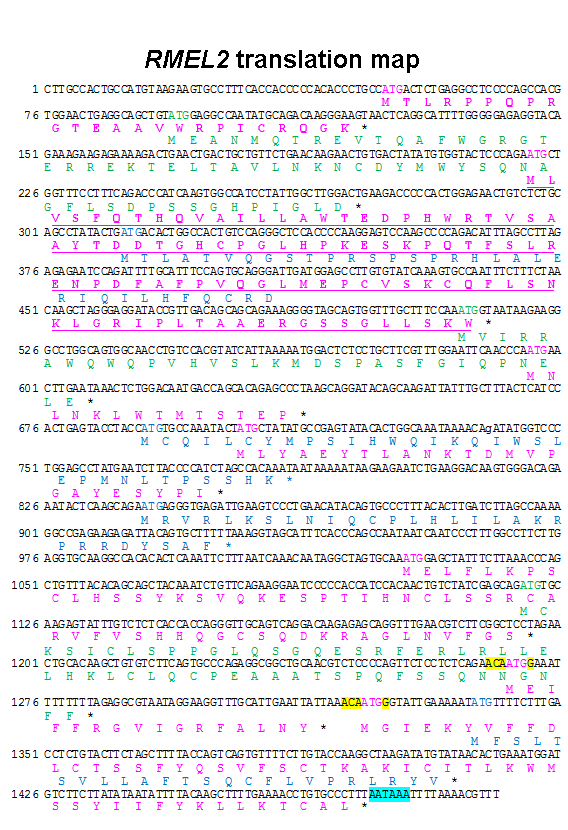


**Figure S2: Putative ORFs and deduced polypeptide sequences for *RMEL2*.** Nucleotide sequence of the longest cDNA sequence and amino acid sequences deduced from all AUG-starting ORFs in the three possible frames (blue, green and pink) using the Tanslation Map tool (<http://www.bioinformatics.org/sms2/> ) and the longest amino acid sequence deduced from a non-AUG-starting ORF (red), detected by Virtual Ribosome tool (<http://www.cbs.dtu.dk/services/VirtualRibosome/>) . The longest deduced amino acid sequence conserved in the other primates is underlined. Sequences highlighted in yellow correspond to Kozac consensus.
