# Supplementary material for: Novel Primate-Specific Genes, RMEL 1, 2 and 3, with Highly Restricted Expression in Melanoma, Assessed by New Data Mining Tool
Source: PLoS One. 2010 Oct 20;5(10):e13510. doi: 10.1371/journal.pone.0013510 (PMC2958148; doi:10.1371/journal.pone.0013510)
Supplement: Figure S4 — A single base pair mutation creates a putative human lineage-specific ORF in RMEL1 gene. (0.14 MB DOC) [file pone.0013510.s008.doc]

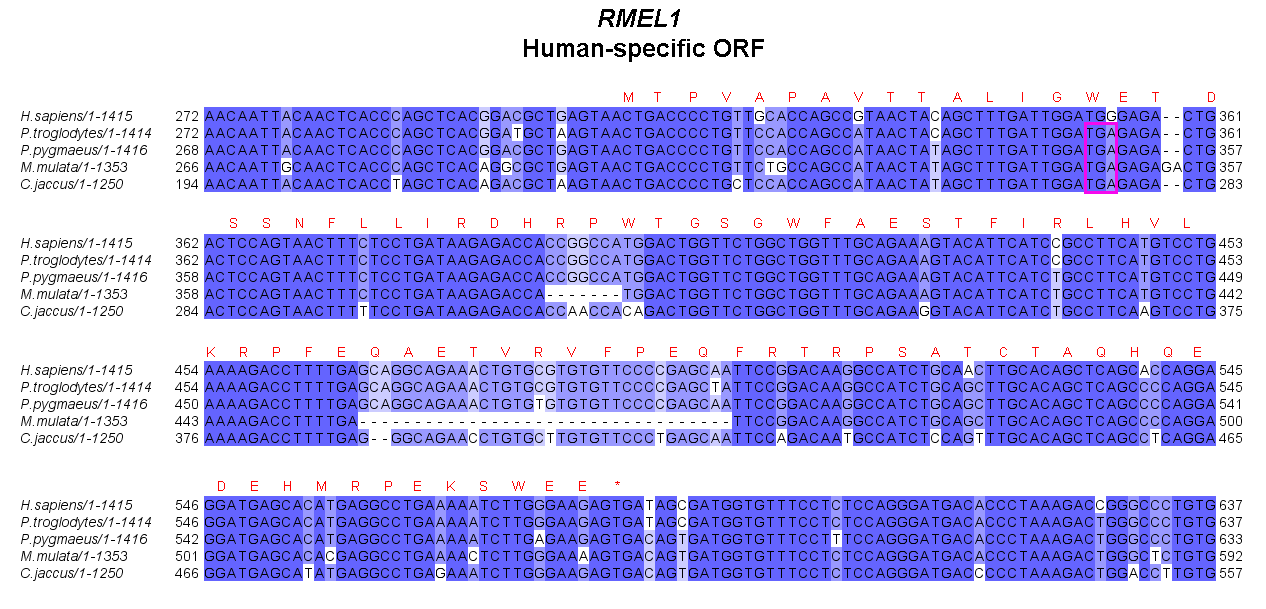


**Figure S4: A single base pair mutation creates a putative human lineage-specific ORF in *RMEL1* gene.** Multiple alignments, generated using ClustalW, of a portion of the expressed sequences of *RMEL1* from five primate species. The alignment comprises a putative human-specific ORF and depicts the region in which the human sequence has c.45A>G substitution that creates a UGG codon for tryptophan in place of a UGA termination codon (pink box) present in the other primate sequences. The deduced amino acid sequence for human *RMEL1* is shown in red, with the aa symbols aligned with the first position of the codon.
